# Cinobufagin as a Potential Intervention Against Liver Cancer—A Comprehensive Review

**DOI:** 10.3390/ph19010158

**Published:** 2026-01-15

**Authors:** Nicole Simone de Lima Coelho, Victória Dogani Rodrigues, Otávio Simões Girotto, Renato César Moretti Júnior, Vítor Engrácia Valenti, Maria Angélica Miglino, Mônica Duarte da Silva, Caio Sérgio Galina Spilla, Ana Luiza Decanini Miranda de Souza, Sandra Maria Barbalho, Lucas Fornari Laurindo

**Affiliations:** 1 Department of Biochemistry and Pharmacology, School of Medicine, Faculdade de Medicina de Marília (FAMEMA), Marília 17519-030, SP, Brazil; 2 Department of Biochemistry and Pharmacology, School of Medicine, Universidade de Marília (UNIMAR), Marília 17525-902, SP, Brazilcaiosgspilla@outlook.com (C.S.G.S.);; 3 Systematic Reviews and Meta-Analyses Center, School of Philosophy and Sciences, São Paulo State University, Marília 17525-900, SP, Brazil; vitor.valenti@unesp.br; 4 Laboratory for Systematic Investigations of Diseases, Department of Biochemistry and Pharmacology, School of Medicine, Universidade de Marília (UNIMAR), Marília 17525-902, SP, Brazil; 5 Graduate Program in Structural and Functional Interactions in Rehabilitation, School of Medicine, Universidade de Marília (UNIMAR), Marília 17525-902, SP, Brazil; 6 Graduate Program in Anatomy of Domestic and Wild Animals, Faculty of Veterinary Medicine and Animal Science, Universidade de São Paulo (FMVZ/USP), São Paulo 05508-270, SP, Brazil; 7 Department of Biochemistry and Nutrition, School of Food and Technology of Marília (FATEC), Marília 17500-000, SP, Brazil; 8 Division of Cellular Growth, Hemodynamic, and Homeostasis Disorders, Graduate Program in Medical Sciences, Faculdade de Medicina, Universidade de São Paulo (USP), São Paulo 01246-903, SP, Brazil

**Keywords:** cinobufagin, bufadienolide, liver cancer, hepatocellular carcinoma, apoptosis, migration, malignancy

## Abstract

Liver cancer remains a significant global health challenge, with hepatocellular carcinoma (HCC) being the most prevalent form. Despite advancements in treatment, high recurrence rates and the limited efficacy of conventional therapies highlight the need for novel interventions. Cinobufagin (CB), a bufadienolide extracted from the parotid secretion of *Bufo gargarizans* and *B. melanostictus*, has emerged as a promising compound with multiple antitumor mechanisms. This comprehensive review assesses the current evidence regarding CB and its containing medicine, cinobufacini, in liver cancer models. Cinobufacini is a traditional Chinese medicine extract, whereas CB refers specifically to one of its active components. The pharmacodynamic actions of CB include induction of apoptosis, DNA damage, inhibition of proliferation and migration, and modulation of key oncogenic pathways such as PI3K/Akt/mTOR, Akt/ERK, and AURKA-mTOR-eIF4E. Additionally, CB disrupts tumor metabolism and induces oxidative stress. Preclinical studies, both in vitro and in vivo, demonstrate significant antitumor efficacy. However, concerns remain regarding CB’s toxicity profile at high doses. This review emphasizes the therapeutic potential of CB in HCC treatment and advocates for further translational research to optimize its clinical applicability, dosage, and safety.

## 1. Introduction

Cancer is a pathology caused by the abnormal proliferation of cells with the potential to reach a metastatic stage. The etiology of this disease is complex, with genetic and epigenetic factors considered key contributors to its development [1]. This condition is associated with the formation of a structurally and functionally abnormal vascularization, promoting localized hypoxia and driving metabolic reprogramming, including increased uptake of extracellular metabolites and elevated metabolic enzyme activity [2]. Considering all types of cancer known worldwide, the one that affects the liver is one of the most common malignancy, given that hepatocellular carcinoma (HCC) is commonly associated with chronic liver diseases, for instance, hepatitis virus infections, notably B and C [3,4]. Among the causes of death in cirrhotic patients, HCC has been recognized as the leading one, and its incidence is expected to increase [5,6,7].

HCC accounts for most primary liver cancers and typically emerges in the context of chronic hepatic inflammation, fibrosis, and cirrhosis. Its development involves progressive genetic and signaling disruptions that promote uncontrolled proliferation, angiogenesis, and resistance to cell death [8,9]. Despite the availability of therapeutic approaches such as surgical resection [10], ablation procedures [11], systemic therapies [12], and targeted agents [13], the prognosis for advanced HCC remains poor, especially when HCC is diagnosed at an advanced stage. In these scenarios, curative options, including surgical resection, liver transplantation, or ablation therapies, are no longer applicable, which underscores the urgent need for new therapeutic strategies [14].

Current conventional treatments for HCC—such as surgical resection and local ablative therapies—are substantially limited by high recurrence rates, which are associated with increased tumor aggressiveness, presence of multinodular disease, large cancer size (≥5 cm), macroscopic vascular or microscopic lymphovascular invasion, high preoperative alpha-fetoprotein levels, and patient-related risk factors (e.g., presence of cirrhosis) [15]. Chemotherapeutic drugs, such as sorafenib, also pose challenges due to issues like toxicity and/or drug inefficacy under long-term use [16]. Therefore, these approaches often fail to provide long-term disease control, particularly in advanced HCC, and many patients remain unsuitable due to comorbidities [17,18]. In this context, natural compounds and phytochemicals emerge as promising alternatives or adjuvant agents, as they tend to exhibit multitarget actions (e.g., inducing apoptosis, inhibiting proliferation/angiogenesis, modulating the immune response) and often show lower toxicity than classical chemotherapeutics [19,20,21,22]. Natural-derived agents like cinobufagin (CB) benefit from these attributes: as a bufadienolide with demonstrated ability to inhibit key oncogenic signaling pathways [e.g., phosphoinositide 3-kinase (PI3K)/protein kinase B (Akt) and mitogen activated protein kinase (MAPK)/extracellular signal-regulated kinase (ERK)], impair angiogenesis, and suppress migration and invasion, CB represents a compound capable of overcoming some of the major drawbacks of conventional therapy—namely tumor recurrence, metastasis, and systemic side effects—thereby supporting its further investigation in the context of HCC [23,24,25,26]. These mechanisms are particularly relevant for HCC, a cancer strongly driven by dysregulated PI3K/Akt and MAPK/ERK signaling [27], making CB a promising candidate for overcoming metastasis and treatment resistance.

With the emergence of new pharmacological therapies, a promising substance, a bufadienolide inhibitor of the sodium–potassium pump, has garnered significant attention in oncology research: CB. This compound is present in the parotid gland secretion of the toad species *Bufo gargarizans* Cantor or *B. melanostictus* Schneider, a fluid called Venenum bufonis. Traditionally used in Chinese medicine, Venenum bufonis is collected from dried toad venom, and CB can be isolated through solvent extraction—commonly ethanol—followed by purification techniques such as high-performance liquid chromatography to obtain CB with high purity [28,29,30]. In addition to CB, another clinically relevant derivative obtained from *B. gargarizans* Cantor is cinobufacini (also called HuaChanSu), a purified aqueous extract widely used in traditional Chinese medicine. Cinobufacini, a mixture of bufadienolides (including CB) and other components, has been developed into injectable and oral formulations [31,32]. It has demonstrated antitumor effects in various malignancies, including HCC, primarily through the induction of apoptosis and suppression of cancer cell migration and invasion [33]. Mentioning cinobufacini is important because CB is one of its major active compounds, and understanding their relationship provides a clearer context for the pharmacological relevance of CB in liver cancer treatment.

When isolated, it demonstrates potential to disrupt the cell cycle, induce apoptosis and deoxyribonucleic acid (DNA) damage, alleviate cancer pain, inhibit the proliferation, migration, and invasion of cancerous cells, and reduce angiogenesis, thereby heightening interest from the scientific community [28,34,35]. Scientific studies have shown that CB downregulates Akt and ERK pathways, which are involved in cell proliferation and migration. This substance becomes even more relevant when considering that recurrence and metastasis are the primary challenges in treating HCC, and that CB has the capability to inhibit proliferation and migration, primarily through its action on the Akt and ERK pathways [36]. The relevance of this study lies in its being the first comprehensive review addressing this specific topic, aiming to provide an overview of the current landscape regarding the results obtained from recent studies on the use of CB in liver cancer treatment. Therefore, this study is innovative in analyzing and correlating the findings regarding CB’s role in treating liver cancer. This paper presents the results obtained up to the time of writing, as demonstrated in the existing literature on this subject. It explores its clinical applicability, considering the dosage, mechanism of action, side effects, and gaps that still require investigation.

**Figure 1:**
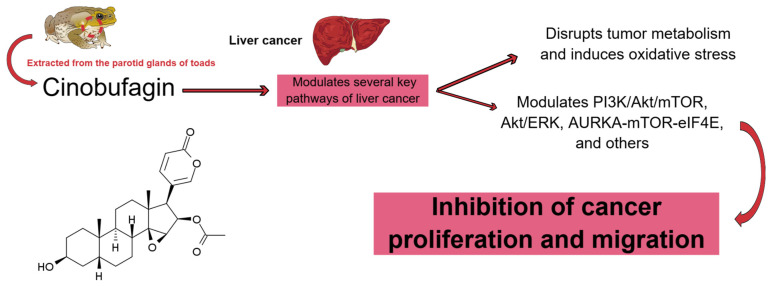
Mechanism of action of cinobufagin in liver cancer. Cinobufagin, a bufadienolide compound extracted from the parotid glands of toads, targets multiple signaling pathways implicated in liver cancer progression. The compound disrupts tumor metabolism and induces oxidative stress while modulating key oncogenic pathways, such as PI3K/Akt/mTOR, Akt/ERK, and AURKA-mTOR-eIF4E. These combined activities contribute to the inhibition of cancer cell proliferation and migration. Created using Mind the Graph (https://mindthegraph.com/, last accessed in December 2025).

As shown in Figure 1, CB—an active compound extracted from the parotid glands of toads—modulates key oncogenic pathways in liver cancer, ultimately suppressing tumor proliferation and migration.

Liver cancer is a global threat. Recently, research has focused on testing animal-derived compounds against liver malignancies due to the current lack of a unique treatment for all stages of the disease. The following sections will explore the potential of CB in combating the development and progression of liver cancer. Firstly, a review of HCC, the most common liver cancer, will be presented. Following this, we will review the most prominent studies on CB and cinobufacini aimed at fighting the development and progression of liver cancer.

## 2. Literature Search Methodology

To investigate the potential of CB in combating liver cancer, we conducted a comprehensive literature search across reputable databases, including PubMed, EMBASE, Cochrane, Scopus, and SpringerLink. The search strategy utilized keywords such as “Cinobufagin,” “Cinobufacini,” “HuaChanSu,” “Liver cancer,” “Hepatocellular carcinoma,” “Molecular mechanisms,” and “Anticancer properties” to ensure rigor and consistency in the findings included in our reports. The inclusion criteria comprised preclinical cellular and animal studies and clinical trials. Specifically, studies needed to present data on CB or CB-containing medications against liver malignancies, indicate potential mechanisms of action and molecular implications, and present therapeutic outcomes and potential based on the treatment applied. Additionally, the studies had to use robust experimental designs and report transparent methodologies and results. Exclusion criteria comprised systematic reviews and meta-analyses, comprehensive reviews, poster presentations, abstract proceedings, conference proceedings, and papers published in a language other than English unless a translation was available. N.S.d.L.C., R.C.M.J., and O.S.G. extracted data using a standardized form to ensure consistency. Information on the administration of the remedy, including dose, route, and duration, as well as the outcomes measured and any limitations, was reported. The quality of each study was assessed based on its sample size, clarity of findings presentation, and robust adherence to guidelines for scientific quality. All the above-mentioned relevant data were synthesized qualitatively, aimed at summarizing the effects of CB across various clinical and preclinical animal and cellular models of liver malignancies, identifying common findings, and discussing any potential limitations. This approach also aimed to highlight gaps in current knowledge and propose directions for future studies.

## 3. Hepatocellular Carcinoma: Pathophysiology, Clinical Features, Diagnosis, Treatment, and Prognosis

**Figure 2:**
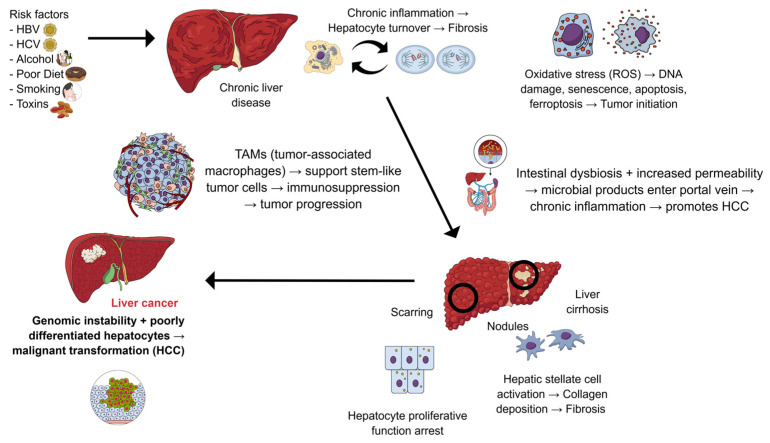
Pathophysiology of hepatocellular carcinoma (HCC). Major risk factors—including chronic infection with hepatitis B virus (HBV) and hepatitis C virus (HCV), alcohol consumption, poor diet, smoking, and exposure to environmental toxins—induce chronic liver injury. Persistent hepatic inflammation increases hepatocyte turnover and activates hepatic stellate cells, resulting in collagen deposition, fibrosis, and cirrhosis with the formation of regenerative nodules. Oxidative stress and reactive oxygen species (ROS) promote DNA damage, senescence, apoptosis, and ferroptosis, contributing to tumor initiation. Disruption of the gut–liver axis through intestinal dysbiosis and increased gut permeability allows microbial products to enter the portal circulation, amplifying inflammation and hepatocarcinogenesis. The tumor microenvironment, particularly tumor-associated macrophages (TAMs), supports stem-like tumor cells, induces immunosuppression, and facilitates tumor progression. Accumulation of genomic instability and poorly differentiated hepatocytes ultimately leads to malignant transformation and development of HCC. Created using Mind the Graph (https://mindthegraph.com/, last accessed in December 2025).

HCC represents the most prevalent form of primary liver cancer and ranks among the most common malignancies worldwide, affecting both men and women. However, men are approximately three times more likely to develop the disease compared to women [37]. According to data from the American Cancer Society (ACS), in 2025, it was estimated that over 42,000 new cases of liver cancer would be diagnosed in the United States, with approximately 30,000 deaths attributed to the disease. The five-year relative survival rate is approximately 20%, being strongly influenced by the stage at which the tumor is diagnosed [38]. In the United States, HCC shows one of the fastest-growing incidence rates among cancers, a trend attributed to modifiable risk factors such as excessive alcohol consumption, smoking, and chronic infection with hepatitis B virus (HBV) and hepatitis C virus (HCV) [39,40,41]. The pathophysiological mechanisms leading from chronic liver injury to HCC are illustrated in Figure 2, which integrates major risk factors, cirrhosis-associated changes, oxidative and inflammatory pathways, the gut–liver axis, and the role of the tumor microenvironment.

Primary liver cancer can be histologically classified into two main types: HCC, accounting for approximately 75% of cases, and intrahepatic cholangiocarcinoma (iCCA), which represents around 15% [42,43]. In most cases, HCC arises in a liver already compromised by chronic liver disease or cirrhosis, with its etiology strongly influenced by regional and individual factors, such as viral infections and exposure to environmental toxins [43,44,45].

The hepatic tumor microenvironment is characterized by substantial infiltration of tumor-associated macrophages (TAMs), which interact with both tumor and immune cells, promoting disease progression. During the early stages of carcinogenesis, TAMs can support tumor stem cells, while the tumor cells, in turn, influence macrophage polarization and activity. As the tumor progresses, TAMs undergo phenotypic changes and emerge as potential therapeutic targets through either the inhibition of pro-tumorigenic markers or functional reprogramming [42].

The liver and gastrointestinal system maintain a close functional interaction through the gut–liver axis, primarily mediated by the portal vein. This system transports not only nutrients and metabolites but also commensal microorganisms and their derivatives directly from the intestine to the liver. Under physiological conditions, the integrity of the intestinal barrier limits the translocation of potentially toxic or pro-inflammatory substances. However, conditions such as liver diseases, chronic alcohol use, antibiotic exposure, and dietary changes can lead to intestinal dysbiosis and compromise of the mucosal barrier [46,47]. Increased permeability allows microbial components to enter the hepatic portal circulation, thereby fostering chronic inflammation, exacerbating liver injury, and potentially contributing to the development of hepatic carcinogenesis. This process also affects immune regulation in both organs, disrupting intestinal and hepatic immune homeostasis [48].

***Oxidative Stress and Tumorigenesis***

Oxidative stress plays a crucial role in tumor initiation and progression. It is defined by an imbalance between reactive oxygen species (ROS) and the cellular antioxidant defense system. This process affects multiple stages of hepatocarcinogenesis, including cellular senescence, apoptosis, and ferroptosis, which occurs through mitochondrial stress, endoplasmic reticulum stress, and DNA damage. Increasing evidence suggests that mitochondria play a central role in regulating these pathways, which can either promote or suppress tumor development, depending on the cellular and microenvironmental context [49].

***Diagnosis***

Early diagnosis is essential for a more favorable prognosis. Imaging modalities such as ultrasound, computed tomography (CT), and magnetic resonance imaging (MRI), in combination with serum alpha-fetoprotein levels, are key diagnostic tools. Biopsy may be employed in cases of diagnostic uncertainty, but it is not always necessary when clinical and radiological criteria are sufficient [50,51].

***Treatment***

Treatment of HCC is determined by the disease stage and hepatic functional reserve. In early stages, curative options include surgical resection, liver transplantation, and radiofrequency ablation. In more advanced stages, systemic therapies become the mainstay. Sorafenib, a tyrosine kinase inhibitor, was the first approved drug for advanced HCC. Still, new therapies are emerging, such as immune checkpoint inhibitors (nivolumab, atezolizumab) and combination therapies with bevacizumab [an anti-vascular endothelial growth factor (VEGF) agent] [51,52]. Emerging therapeutic strategies include the use of genetically engineered macrophages expressing chimeric antigen receptors (CAR-M) aimed at immune reprogramming of the tumor microenvironment, as well as targeting specific receptors on TAMs. These approaches offer promising alternatives for the treatment of both HCC and iCCA [44].

Animal medicines have also been described as an alternative emerging option against various diseases [53]. CB, cinobufotalin, bufotalin, and bufalin from *B. gargarizans* Cantor or *B. melanostictus* Schneider, gepsin from *Gekko swinhonis*, cantharidin and norcantharidin from *Mylabris phalerata* Pallas or *Mylabris cichorii* Linnaeus, beauvericin from *Bombyx mori* Linnaeus, sipholenol A from *Siphonochalina*, etc., are all animal-derived molecules aimed at fighting against liver cancers via various mechanisms, which involve apoptosis induction, anti-proliferation, overcoming drug resistance, anti-migration, and autophagy induction [54].

***Prognosis***

The prognosis of HCC remains challenging. Survival is mainly dependent on early detection and eligibility for curative-intent therapies. The presence of cirrhosis, vascular invasion, and metastases is associated with poorer outcomes. Nevertheless, recent therapeutic advances have improved survival even in advanced stages of the disease [43,44,55].

## 4. Biosynthesis of Cinobufagin in the Toad’s Gland

Bufadienolides are a class of steroidal compounds characterized by a steroid nucleus bearing a six-membered α-pyrone ring at the 17-position carbon, encompassing CB. This substance plays a role in defending amphibians against potentially lethal fungal and bacterial infections. Strategies involving nanoparticles have been used to understand the potential of amphibian toxins. Since bufadienolides exhibit relatively low solubility, their efficacy has been enhanced by encapsulating them in nanoparticles with hydrophobic components, such as liposomes [56]. In addition to improving uptake by cells and the delivery to the tumor sites, liposome encapsulation has been shown to reduce the inherent side effects in vivo of CB by enabling sustained drug release and enhanced anticancer efficacy, providing a membrane-mimicking environment that allows researchers to study its mechanism of cancer disruption under safer and more physiologically relevant conditions [57,58]. The bufadienolides class has steroids as precursors. However, despite numerous studies aimed at identifying the exact substance that initiates the production of this class of compounds, this information remains unknown. Therefore, further research is necessary to elucidate the most probable biosynthetic pathway [59]. Existing evidence suggests that bufadienolides likely arise from cholesterol-derived steroid precursors through a series of modifications, including hydroxylation and formation of the characteristic six-member lactone ring. Enzymes such as cytochrome P450s are suspected to participate in this pathway [60]. A more precise identification of the precursor molecules and the sequence of enzymatic steps would help define a complete biosynthetic map for CB.

## 5. Pharmacodynamics and Pharmacokinetics of Cinobufagin

### 5.1. Pharmacodynamics

CB can induce apoptosis, a form of programmed cell death [61]. In this context, this substance can cause this process by activating caspases via both the death receptor pathway and the mitochondrial apoptosis pathway [62]. Some signaling pathways modulated by CB include Lymphoid Enhancer-Binding Factor 1 (LEF1), MAPK, Signal Transducer and Activator of Transcription 3 (STAT3), and PI3K/Akt [63]. It has been observed that CB increases the expression of γ-H2AX, a histone family protein that accumulates in the cell nucleus and indicates the presence of DNA damage. Another noteworthy property of CB is its ability to suppress the activity of the enzyme thymidylate synthase (TYMS), which is responsible for catalyzing the methylation of deoxyuridine monophosphate (dUMP) and, consequently, converting this nucleotide into deoxythymidine monophosphate (dTMP), also known as thymidylate, a molecule essential for DNA synthesis. This ability of CB is of great significance, given that this reaction is crucial for maintaining and stabilizing nucleotide pools during cellular growth and development [64].

Another capability of CB is to inhibit cancer cell proliferation by exerting effects on the expression of the epidermal growth factor receptor (EGFR) in various cancer types. CB also attenuates phosphorylation of ERK and the expression of c-Myc [34,65]. Considering that both EGFR and c-Myc are proto-oncogenes—commonly occurring genes susceptible to alterations that may transform them into oncogenes—the action of CB on these targets directly impacts cellular proliferation. Additionally, at sublethal doses, CB generates an overload of ROS, resulting in significant DNA damage and oxidative stress, which in turn induce apoptosis [34].

CB exerts its antitumor activity in HCC by simultaneously triggering apoptosis and modulating oncogenic signaling. CB’s inhibition of TYMS further compromises DNA integrity and synthesis, creating lethal replication stress in HCC cells. Additionally, the downregulation of Akt/ERK pathways impairs proliferative signaling essential for liver cancer progression. CB also increases ROS, reducing HCC cells viability. Together, these mechanisms clarify how CB’s modulation of multiple interconnected processes ultimately converges on reduced proliferation, elevated oxidative stress, and apoptosis in liver cancer cells.

### 5.2. Pharmacokinetics

The primary reaction involved in the metabolism of CB is hydroxylation, which appears in the bile [34]. In an experimental model conducted with rats, it was demonstrated that the biotransformation of CB results in the formation of the metabolites desacetylcinobufagin and epidesacetylcinobufagin. Evidence suggests that the desacetylation of CB occurs in both the plasma compartment and hepatic tissue. When the intensity of this reaction is compared between rats and rabbits, a more pronounced enzymatic activity is observed in rabbits. Additionally, in a separate experimental setup, it was observed that both desacetylation and epimerization of CB take place in the liver, underscoring the central role of this organ in the metabolic modification of the compound. In rats, the conversion of CB into 3-keto-desacetylcinobufagin was found to be catalyzed by the enzyme β-hydroxysteroid dehydrogenase, with the intermediate subsequently being transformed into epidesacetylcinobufagin via the action of 3α-hydroxysteroid dehydrogenase. Notably, under the experimental conditions, no measurable concentrations of the metabolites mentioned above were detected in the analyzed tissues, suggesting either rapid metabolism or elimination, or possible methodological limitations related to the analytical sensitivity employed [66].

In terms of elimination, current evidence indicates that CB and its major metabolites are predominantly cleared through biliary excretion. The detection of hydroxylated and desacetylated metabolites primarily in bile, rather than in systemic tissues, suggests rapid hepatic processing followed by direct transport into the biliary tract for fecal elimination [34]. However, trace amounts of CB metabolites have been reported in urine, indicating that renal elimination can also play a role [67]. The majority of the evidence supports the notion that CB undergoes swift hepatic biotransformation coupled with efficient biliary clearance [68]. Together, these findings clarify that CB is eliminated mainly via hepatic metabolism followed by biliary excretion, consistent with its strong liver-directed pharmacokinetic profile.

In summary, CB undergoes extensive hepatic biotransformation, primarily through hydroxylation, desacetylation, and epimerization reactions that occur within liver tissue. The formation of metabolites such as desacetylcinobufagin and epidesacetylcinobufagin underscores the central metabolic role of hepatocytes, while the detection of these metabolites predominantly in bile indicates rapid processing and elimination via hepatic pathways. This strong liver-directed metabolism is particularly relevant for HCC, as it may influence local drug exposure within the tumor microenvironment. Collectively, the metabolic profile of CB supports its pharmacological relevance in liver cancer by linking its biotransformation directly to the hepatic function, the primary site of both metabolism and therapeutic action.

## 6. Physicochemical Properties

**Figure 3:**
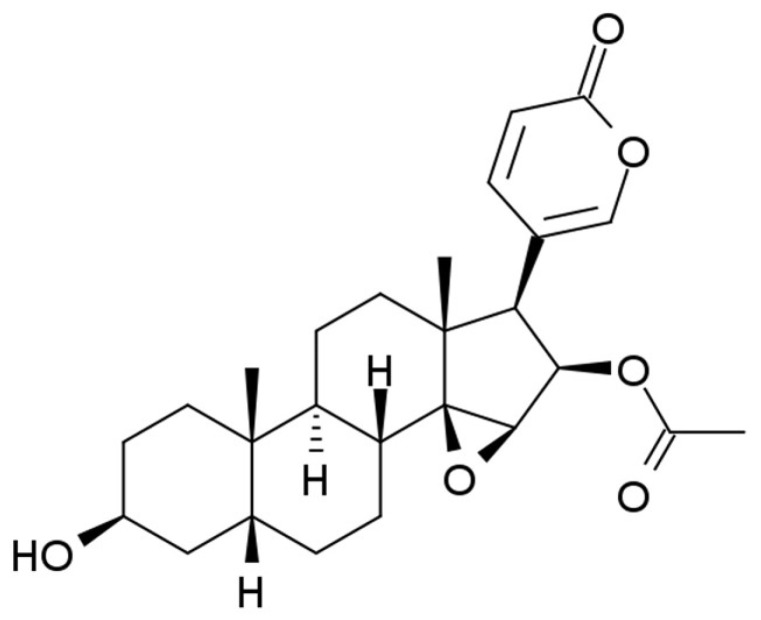
Molecular structure of cinobufagin following Wang et al. [70].

CB is characterized by the molecular formula C_26_H_34_O_6_, molecular weight of 442.54 g/mol, and melting point of 222–223 °C [35]. Its topological polar surface area (TPSA) is 85.4 Å^2^, corresponding to an XLogP3-AA value of 3.3, classifying CB as a moderately lipophilic compound. It is classified within the lipid category, specifically under the subclass of steroids [69]. Figure 3 illustrates the molecular structure of CB following the study conducted by Wang et al. [70].

The physicochemical characteristics of CB have direct implications for its biological activity and pharmacokinetic behavior. Its poor water solubility, as well as its short circulating half-life, promotes low bioavailability. However, the lipophilicity facilitates simple diffusion across biological membranes, supporting its absorption in lipid-rich environments—an important factor for steroid-like compounds that interact with membrane-bound targets [71]. The physicochemical properties also suggest that CB’s structure allows permeability for oral absorption [72].

Additionally, its steroidal framework contributes to the high affinity for Na^+^/K^+^-ATPase, the primary molecular target of bufadienolides, by promoting stable hydrophobic contacts within the enzyme’s binding pocket. However, CB’s structure presents polar substituents on ring D of the steroid core. This may interfere with the electrostatic interactions between the lactone ring and K^+^ in the cation binding site [73,74]. These structural and physicochemical features may influence CB’s distribution, ultimately favoring penetration into tissues with high membrane content and supporting its broad spectrum of biological actions, including cardiotonic and anticancer effects [75]. Thus, the physicochemical properties of CB are integral to understanding its mechanism of action, membrane permeability, and tissue distribution profiles.

## 7. Toxicity and Safety

Another essential aspect to recognize is that, at high doses, CB becomes toxic to the human body, triggering cardiac arrhythmias, seizures, muscle spasms, or paralysis. Additionally, it may cause heart failure through acute local ischemia. As it becomes toxic, CB induces poisoning symptoms, such as eyelid closure and respiratory depression [34].

CB exhibits analgesic effects at safe concentrations by acting on peripheral opioid receptors. In addition, the different impacts of CB observed include antifibrotic activity, which suppresses the activation and differentiation of fibroblasts, as well as antiviral and antiprotozoal properties [76].

It is considered a toxic substance, being potentially fatal in cases of ingestion, inhalation, or dermal contact. In acute toxicity studies conducted in mice, oral administration of a dose of 144 mg/kg resulted in alterations to sensory organs (e.g., the eyes, with manifestations such as ptosis), general activity (notably central nervous system depression), and behavior (including ataxia). In contrast, intravenous administration at a 1210 µg/kg dose in mice elicited behavioral changes characterized by tremors and seizures or effects on the seizure threshold [69].

To minimize the risk of CB-induced toxicity, several strategies must be investigated. First, dose optimization and rigorous therapeutic window determination are essential, as bufadienolide compounds exhibit toxicity by inhibiting the Na^+^/K^+^-ATPase, which is a critical target involved in their pharmacological and toxic impacts and widely distributed in various cells of the human body [77,78,79]. Controlled-release formulations have been proposed to achieve therapeutic concentrations [80,81]. Additionally, structural modification and semisynthetic derivatives of CB are being explored to improve solubility and therapeutic activity. However, these approaches may raise other safety concerns [82]. At the clinical level, co-administration with cardioprotective agents or compounds that modulate Na^+^/K^+^-ATPase signaling may also attenuate arrhythmogenic effects. Proper monitoring of the cardiac function, electrolytes, and neurological symptoms may be recommended when CB is administered experimentally or therapeutically. Finally, standardized purification and quality-control procedures may help eliminate contaminants that might exacerbate toxicity. Collectively, these approaches highlight that CB’s therapeutic potential may need to be balanced with appropriate safety strategies.

## 8. Assessing the Potential of Cinobufagin and Cinobufacini in Combating Liver Cancer Development and Progression

### 8.1. Anticancer Studies of Cinobufagin and Cinobufacini: Mechanisms, Efficacy, and Potential Clinical Implications for Liver Cancer

Table 1 presents the results of seven experimental studies on the effect of CB alone on liver cancer models. In general, the results of all the analyzed studies were positive, with mechanisms of action ranging from the induction of apoptosis and identification of metabolic vulnerabilities to the inhibition of cell proliferation. Table 2 presents the analysis of 12 studies in which the primary intervention and the main reported effects were associated with cinobufacini/HuaChanSu or its constituent components in liver cancer models. Each experiment will be subsequently discussed based on the study type, intervention, the compound’s mechanism of action under investigation, results, limitations, and potential clinical implications. Table 3 presents two studies investigating the use of cinobufacini/HuaChanSu in clinical trials.

#### Literature Search Report

**Figure 4:**
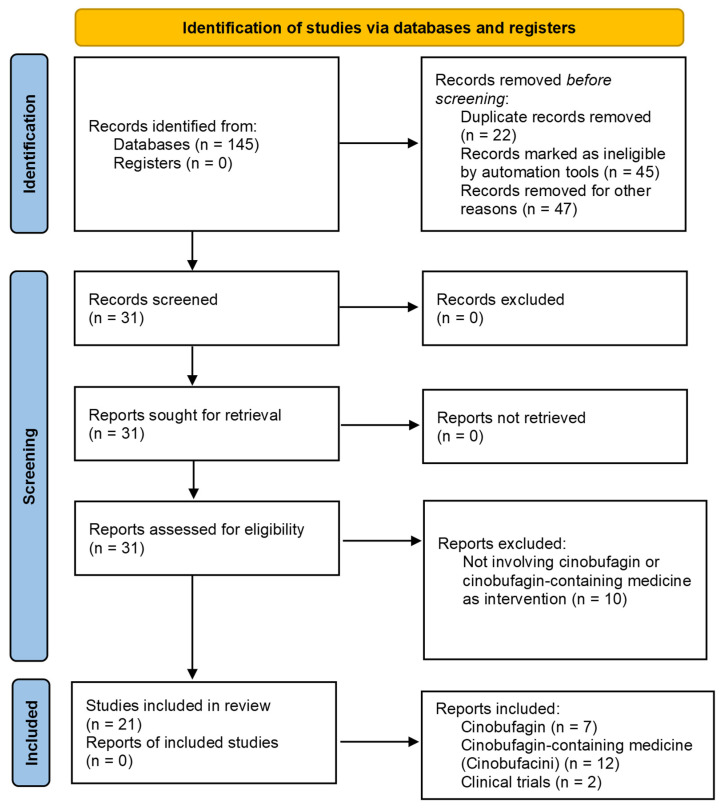
PRISMA Flow Diagram depicting the study search and selection process.

Figure 4 depicts, based on the Preferred Reporting Items for Systematic reviews and Meta-Analyses (PRISMA) Guidelines, the flow diagram of study search and selection. Initially, 145 studies were identified from databases. At this stage, 22 records were removed due to duplication, 45 records were marked as ineligible by automation tools, and 47 records were excluded due to inconsistencies, such as a language other than English and review papers. Following this, 31 records were screened and retrieved. At this stage, 10 reports were excluded due to the lack of involvement of CB or a CB-containing drug as an intervention. Finally, 21 studies were included in the final analysis: 7 reporting results of preclinical studies on CB alone against liver cancer, 12 reporting the results of cinobufacini or HuaChanSu and its components, and 2 reporting clinical trials.

### 8.2. Assessing Cinobufagin Alone in Intervening with Liver Cancer Models

The first investigation involved treating HepG2 and Huh-7 cell lines in an in vitro experimental model. It was observed that the mechanism of action of CB involved the activation of cuproptosis, a copper-dependent form of apoptosis. The results demonstrated that the compound reduced cell viability and increased the concentration of ROS. As a limitation, this study was conducted solely in vitro and utilized only two cell lines, which restricts the generalizability of the findings and highlights the need for further research to assess its large-scale applicability. Nonetheless, the study stands out for its potential clinical implications, particularly due to its suggested effectiveness against apoptosis-resistant cancer [83].

The second study focused on HepG2 and SK-HEP-1 cell lines treated with CB, based on its mechanism of action, which involves the disruption of lipid, amino acid, carbohydrate, and nucleotide metabolism. As a result, the compound demonstrated the ability to inhibit cell proliferation and alter cellular metabolism. It is essential to note the limitation of this study, as it was conducted solely in vitro and employed only two cell lines. Nevertheless, the potential of this compound to target metabolic vulnerabilities in tumor cells is a highly relevant finding, warranting further investigation [84].

The third study involved the treatment of the HepG2 and Huh-7 cell lines and HepG2 nude mouse model with CB. The observed mechanism of action included inhibition of the PI3K/Akt/mammalian target of rapamycin (mTOR) signaling pathway. This intervention resulted in reduced proliferation of tumor cells and, when combined with the autophagy inhibitor (MRT68921), enhanced apoptosis. A limitation of this study is that it was conducted using only two cancer cell lines. Nonetheless, the study opens up avenues for exploring whether the combination of CB with autophagy-inhibiting agents can enhance its therapeutic efficacy [85].

The fourth study was an in vitro experimental investigation involving the application of varying concentrations of CB to the HepG2 cell line. The study assessed the drug’s ability to activate p53, Bcl-2-associated X protein (Bax), and caspase-3, as well as to inhibit the Akt/ERK signaling pathway. The results demonstrated that the compound induced apoptosis and inhibited both cell proliferation and migration. It is essential to acknowledge the study’s limitations, as it was conducted solely in vitro and involved only a single cell line. Nevertheless, its significance lies in the potential of the experiment to pave the way for further investigation into the applicability of CB, particularly given its prospective mechanism of action via the Akt/ERK pathways [36].

The fifth study involved treating HepG2 and SK-HEP-1 cell lines with CB. The investigation was based on the substance’s potential to degrade TYMS and induce DNA damage and cell cycle arrest. The results indicated inhibition of cell proliferation and colony-forming ability. Although the study has limitations—being conducted exclusively in vitro and involving only two cell lines—it demonstrates that CB holds potential as a therapeutic agent targeting the TYMS enzyme [64].

The sixth study involved treating the Huh-7 cell line and a Huh-7 xenograft mouse model with CB, while modulating Aurora kinase A (AURKA) expression through both overexpression and inhibition, to evaluate the role of this protein in the compound’s mechanism of action. The study was based on the premise that inhibition of the AURKA–mTOR–eukaryotic translation initiation factor 4E (eIF4E) axis impacts translation and mitotic processes. The results revealed mitotic defects, DNA damage, and cell cycle arrest. Although the study had a brief intervention period, it highlights the potential of CB to inhibit cancer cell proliferation by interfering with protein translation [86].

The seventh study involved the application of CB in Huh-7 cells harboring a mutation in the gene encoding the p53 protein. The study was based on the mechanism of CB, which involves AURKA inhibition and p73 activation. The experimental results demonstrated an induction of apoptosis and cell cycle arrest. Although the study was conducted solely in vitro and used only a single cell line, it has promising implications for cancers harboring mutant p53 [87].

Taken together, the studies evaluating CB as a single, isolated compound consistently demonstrate notable antitumor activity across liver cancer cell lines, despite their preclinical nature. Although each investigation targeted distinct mechanistic pathways—including cuproptosis induction, metabolic disruption, inhibition of PI3K/Akt/mTOR and Akt/ERK signaling, TYMS degradation, and suppression of the AURKA-regulated translational and mitotic machinery—all studies converge on the overarching finding that CB suppresses proliferation and promotes apoptosis or cell cycle arrest. However, the uniform reliance on a limited set of cell lines and animal models significantly restricts generalizability, leaving critical questions regarding pharmacodynamics, safety, and clinical applicability unresolved. Nonetheless, the mechanistic diversity observed across these experiments suggests that CB exerts multifaceted anticancer effects and may hold promise as a targeted agent—particularly for tumors displaying metabolic vulnerabilities, AURKA overactivity, defective p53, or resistance to conventional apoptosis pathways—warranting further translational and in vivo research.

### 8.3. Assessing Cinobufagin-Containing Standardized Drug Cinobufacini or HuaChanSu in the Intervention with Liver Cancer Models

The first study was an in vitro experimental investigation that applied cinobufacini to HepG2 and SK-HEP-1 cells. The study was based on the downregulation of TYMS expression and the compound’s interference with DNA synthesis. The results included inhibition of cell proliferation. Key limitations include reliance solely on in vitro experiments. The study’s potential clinical implications suggest the possible use of cinobufacini in combination with chemotherapeutic agents or as a standalone treatment for tumors with TYMS overexpression [88].

The second study was an in vitro/in vivo experimental investigation. Cinobufacini was applied, in different dosages, to HepG2 and Huh-7 cells and to a HepG2 xenograft mouse model. The study was based on the modulation of the c-Met signaling pathway and reported outcomes, including reduced cell proliferation and migration, as well as inhibition of c-Met phosphorylation and the downstream mitogen-activated protein kinase kinase (MEK)/ERK pathways. Its limitations included the lack of human experimentation. Nonetheless, the study suggests that the drug may be a promising candidate for targeting c-Met in advanced HCC [33].

The third study was an in vitro/in vivo experimental investigation. Cinobufacini was injected into a Hepa1-6 xenograft mouse model, and Hepa1-6 cells were treated with a drug-containing serum. The study focused on modulating the sterol regulatory element-binding protein 1 (SREBP1) pathway and macrophage polarization. The results indicated an inhibition of tumor progression, along with alterations in lipid metabolism and the tumor microenvironment. Limitations include the absence of human data and a brief period of intervention. Nonetheless, the study suggests therapeutic potential through metabolic and immune modulation [89].

The fourth study was an in vitro experimental investigation. HuaChanSu was administered to HepG2 and Huh-7 cell lines. The study focused on mechanisms such as a reduction in cell proliferation and viability, through downregulation of glucose-6-phosphate dehydrogenase (G6PD) activity. The main limitation of the study was the absence of in vivo and clinical data. Nonetheless, the experiment highlighted the potential of HuaChanSu as an adjuvant therapeutic strategy for liver cancers [90].

The fifth study was an in vitro/in vivo investigation, employing HepG2 and SK-HEP-1 cell lines and H22 hepatoma-bearing mice. The HuaChanSu was administered, aiming to inhibit glycolysis via suppression of Hexokinase-2. The results demonstrated a reduced tumor volume and cell proliferation. A key limitation of the study is the absence of clinical translation to date. This study suggested promising therapeutic potential by targeting tumor metabolism and growth [91].

The sixth study was an in vitro/in vivo experimental investigation that evaluated the effects of cinobufacini injection and bufothionine, a component of cinobufacini, in a hepatoma-bearing mouse model and in the SMMC7721 cell line. The study was based on inhibition of the Janus kinase 2 (JAK2)/STAT3 signaling pathway and induction of autophagy. The results demonstrated reduced cell proliferation. A key limitation of the study is the lack of clinical data. Nevertheless, the experiment suggests potential for targeting STAT3-mediated signaling [92].

The seventh study, an in vitro experimental investigation, involved the application of cinobufacini to the HepG2 cell line. The study was based on the modulation of the c-Met/ERK pathway and suppression of epithelial–mesenchymal transition (EMT). Results showed reduced cell proliferation, migration, and invasion, as well as downregulation of N-cadherin and vimentin. Although the study was limited to in vitro assays, it suggests a potential treatment strategy for HCC by targeting EMT [93].

The eighth study, an in vitro/in vivo experimental study, administered bufadienolides to the BEL7402 cell line and to HepG2 and H22-tumor bearing mice. The rationale was based on bufadienolides being identified as one of the main active components of cinobufacini. The results confirmed an inhibition of tumor growth. A limitation was the absence of specific assays investigating the mechanism of action. Nevertheless, the study is relevant as it demonstrates the importance of bufadienolide concentration for the therapeutic effects of cinobufacini [94].

The ninth study, an in vitro experimental investigation, applied cinobufacini to HepG2 and HLE cell lines. The study was based on activation of the Fas and mitochondrial apoptotic pathways. The combined treatment with doxorubicin intensified apoptosis compared to each agent alone. The investigation being based only on in vitro experiments is a limitation. However, it suggests that combined treatment via a potential synergistic regimen may be more effective in shaping clinical outcomes for HCC patients [95].

The tenth study, an in vitro experimental investigation, applied cinobufacini to the HepG2 cell line, based on suppression of topoisomerase I and II expression. The study demonstrated a dose-dependent inhibition of cell proliferation. Limitations include the exclusive use of in vitro experiments and the evaluation of only a single cell line. Nonetheless, this study is relevant as it may contribute to improving the existing chemotherapy regimens [96].

The eleventh study, an in vitro experimental study, applied cinobufacini to the HepG2 cell line, focusing on morphological alterations detected by atomic force microscopy. Results showed cytoskeletal destruction and antiproliferative effects. Limitations include the absence of in vivo experiments and the use of only one cell line. The relevance lies in providing an alternative method for early detection of cytotoxic responses [97].

The twelfth study, an in vitro experimental investigation, used an aqueous extract of cinobufacini on HepG2 and BEL7402 cell lines, based on the mitochondria-mediated apoptotic pathway. The results showed an induction of apoptosis via activation of Bax, cytochrome c release, and caspase activation. The lack of in vivo experiments is one limitation of this study. However, its potential clinical significance lies in its role as a classic activator of the apoptotic pathway, improving current anticancer therapies [98].

Overall, the preclinical evidence evaluating cinobufacini/HuaChanSu preparations and their components demonstrates a consistent antitumor effect across diverse liver cancer models. Despite substantial heterogeneity in formulation, concentration, and mechanistic focus, the studies collectively implicate multiple tumor-suppressive pathways, including inhibition of proliferative signaling, disruption of metabolic processes, induction of apoptosis, and modulation of the tumor microenvironment. A challenge is that most investigations remain confined to early-stage preclinical models without human validation. Nonetheless, the cumulative findings indicate that cinobufacini/HuaChanSu preparations and their components exert multifaceted antitumor activity and may hold therapeutic promise both as standalone agents and in combination regimens, warranting further standardized and clinically oriented research.

### 8.4. Assessing Cinobufagin-Containing Standardized Drug Cinobufacini or HuaChanSu in the Intervention with Liver Cancer Within Human Clinical Trials

The first study was a pilot, phase I clinical trial involving eleven patients with advanced HCC. Patients received intravenous cyclic administrations of HuaChanSu at 10, 20, 40, 60, or 90 mL/m^2^ per cycle over 14 consecutive days, followed by a 7-day rest period. No placebo or control group was included. One patient exhibited a 20% reduction in tumor burden after 11 months of administration of 10 mL/m^2^ HuaChanSu, and patients with stable disease reported improvements in quality of life. However, the lack of a control group and the absence of placebo administration limit the internal validity and generalizability of the findings [99].

The second study was a multicenter, randomized, open-label controlled trial that enrolled 364 patients aged 18 to 75 years, all of whom had undergone radical hepatectomy and had histologically confirmed HCC. The intervention group received a cinobufacini injection of 50 mL per day for 10 days every three months over twelve months, in combination with Jiedu granules at a dose of 4.5 g twice daily for six months. The control group was treated with transarterial chemoembolization. The results demonstrated that HuaChanSu, when combined with Jiedu granules, significantly reduced the risk of HCC recurrence following surgical resection, with a similar adverse event profile compared to the control group. Nonetheless, the efficacy of each individual component was not assessed separately, which limits the conclusions drawn [100].

Collectively, the available clinical evidence on CB-containing preparations in HCC remains preliminary but suggests a possible therapeutic value. The early phase I pilot trial demonstrated signals of biological activity and potential quality-of-life benefits, yet the absence of a control arm and the small sample size severely limit conclusions regarding efficacy. In contrast, the larger, multicenter, randomized controlled study provides stronger evidence that cinobufacini—particularly when combined with Jiedu granules—may contribute to reduced postoperative recurrence in patients undergoing radical hepatectomy. However, the open-label design, concomitant herbal intervention, and potential variability in liver function across patients complicate an interpretation of the isolated effects of CB-containing formulations. Taken together, these studies indicate that a clinical benefit is plausible but not yet definitively established, underscoring the need for rigorously controlled, standardized, and formulation-specific trials to determine the true therapeutic role of cinobufacini/HuaChanSu in liver cancer management.

## 9. Conclusions

CB has demonstrated significant antitumor potential against HCC through diverse mechanisms, including the induction of apoptosis, suppression of proliferation and migration, interference with DNA synthesis and repair, and modulation of key oncogenic signaling pathways. Preclinical studies consistently support its efficacy. Only two clinical studies on CB-containing formulations (cinobufacini/HuaChanSu) are available. This limited clinical evidence base constrains the ability to draw robust conclusions regarding efficacy, safety, and broader applicability in diverse patient populations. Despite the scarcity of clinical trials, early findings are promising and indicate improvements in tumor control and recurrence prevention. However, the compound’s toxicity at high doses and the variability in cinobufacini formulations underscore the need for standardized clinical protocols and further safety evaluations. As the first comprehensive review linking CB to liver cancer treatment, this study highlights the therapeutic relevance of this natural compound and reinforces the importance of continued translational research. Future investigations should focus on elucidating precise molecular mechanisms, optimizing dosing regimens, and conducting well-designed clinical trials to validate its clinical utility and expand therapeutic options for patients with HCC.

Taken together, this review emphasizes the need to consolidate current knowledge through more rigorous and harmonized research efforts. While preclinical data provide a strong foundation, the limited and heterogeneous clinical evidence demands a systematic evaluation under standardized conditions. To advance the field, future work should prioritize defining the pharmacological profile of CB in humans, establishing consistent manufacturing and formulation standards, and identifying patient subgroups most likely to benefit. Furthermore, integrating CB into contemporary therapeutic frameworks—such as immunotherapy- or targeted therapy-based regimens—may help clarify its potential role in combination strategies. Addressing these research gaps will be essential for translating CB from a promising experimental agent into a clinically reliable option for HCC.

## Figures and Tables

**Table 1:** Effects of cinobufagin alone in the intervention of liver cancer models: outcomes, limitations, and implications across different studies.

Study’s Type	Cell Line(s)/Tumor Model(s)	Intervention(s)	Mechanism(s) of Action	Outcomes	Limitations	Possible Clinical Implications	Ref.
Experimental in vitro	HepG2 and Huh-7.	Cinobufagin (IC_50_ = 25 nM).	Activates cuproptosis (copper-dependent cell death).	Reduces cell viability, increases ROS.	In vitro only, two cell lines.	Potential in apoptosis-resistant cancer.	[83]
Experimental in vitro	HepG2 and SK-HEP-1.	Cinobufagin (31.25, 50, 62.5, 100, 125, and 500 nM).	Disrupts lipid, amino acid, carbohydrate, and nucleotide metabolism.	Inhibits cell proliferation and alters metabolism.	In vitro only, two cell lines.	Promising by targeting metabolic vulnerabilities.	[84]
Experimental in vitro and in vivo	HepG2 and Huh-7. HepG2 nude mouse model.	Cinobufagin (IC_50_ = 3.8 µmol/L and 3.7 µmol/L for Huh-7 and HepG2 cells, respectively) in vitro. 1 mg/kg in vivo.	Inhibits PI3K/Akt/mTOR and induces autophagy.	Reduces cell proliferation; combining with an autophagy inhibitor (MRT68921) enhances apoptosis.	Two cell lines.	Combination with autophagy inhibitors may improve antitumor efficacy.	[85]
Experimental in vitro	HepG2.	Treatment with varying concentrations (0–320 ng/L) of cinobufagin.	Activates p53, Bax, and caspase-3, inhibits Akt/ERK.	Induces apoptosis, inhibits cell proliferation and migration.	In vitro only, one cell line.	Potential target via Akt/ERK pathways.	[36]
Experimental in vitro	HepG2 and SK-HEP-1.	Cinobufagin (5, 25, 31.25, 50, 62.5, 100, 125, 500, and 1000 nM).	Induces proteasomal degradation of TYMS, induces DNA damage and G2 cell cycle arrest.	Inhibits cell proliferation and colony-forming ability.	In vitro only, two cell lines.	Potential anticancer therapy targeting TYMS.	[64]
Experimental in vitro and in vivo	Huh-7. Xenograft (Huh-7) nude mouse model.	Treatment with cinobufagin (1 μmol/L), and manipulation of AURKA expression (through overexpression and inhibition). Mice were treated with 10 mg/kg of cinobufagin for 21 days.	Inhibits AURKA-mTOR-eIF4E axis and Cap-dependent translation.	Mitotic defects, DNA damage, and G2/M cell cycle arrest.	Brief intervention period.	Potential to block cancer cell proliferation.	[86]
Experimental in vitro	Huh-7 (mutant p53).	Treatment with cinobufagin (IC_50_ = 5.1 µmol/L for 24 h) and manipulation of AURKA.	Inhibits AURKA and activates p73.	Induces apoptosis and G2/M cell cycle arrest.	In vitro only, one cell line.	Promising for cancers with mutant p53.	[87]

**Abbreviations:** Akt—Protein kinase B; AURKA—Aurora kinase A; Bax—Bcl-2-associated X protein; DNA—Deoxyribonucleic acid; eIF4E—Eukaryotic translation initiation factor 4E; ERK—Extracellular signal-regulated kinase; HCC—Hepatocellular carcinoma; mTOR—Mammalian target of rapamycin; PI3K—Phosphoinositide 3-kinase; p53—Tumor suppressor protein p53; p73—Tumor protein p73; ROS—Reactive oxygen species; TYMS—Thymidylate synthase.

**Table 2:** Effects of cinobufagin-containing standardized drug cinobufacini/HuaChanSu and its components in the intervention of liver cancer models: outcomes, limitations, and implications across different studies.

Study’s Type	Cell Line(s)/Tumor Model(s)	Intervention(s)	Mechanism(s) of Action	Outcomes	Limitations	Possible Clinical Implications	Ref.
Experimental in vitro	HepG2 and SK-HEP-1.	Cinobufacini (0.5, 1, 2, 4, and 8 µg/mL).	Downregulates TYMS expression; affects DNA synthesis.	Inhibits cell proliferation.	In vitro only.	May be combined with 5-FU or used in TYMS-overexpressing tumors.	[88]
Experimental in vitro and in vivo	HepG2 and Huh-7. Xenograft (HepG2) nude mouse model.	Cinobufacini (0.025, 0.05, 0.1, 0.2, 0.3, 0.4, and 0.5 mg/mL in vitro; 3 mg/kg in vivo).	Inhibits the phosphorylation of c-Met and the downstream PI3K/Akt and MEK/ERK pathways; decreases MMP-2 and 9 expression; increases Bax/Bcl-2 ratio and caspase-3 cleavage.	Reduces cell proliferation, migration, and invasion; promotes apoptosis.	No human trials.	Candidate for targeting c-Met in advanced HCC and its metastasis.	[33]
Experimental in vitro and in vivo	Hepa1-6. Xenograft (Hepa1-6) mouse model.	Cinobufacini injection (1.17, 2.34, and 3.51 g/kg) in vivo and drug-containing serum in vitro.	Inhibits the AMPK/SREBP1/FASN signaling pathway and M2 macrophage polarization.	Inhibits cell proliferation and tumor progression; alters lipid metabolism and tumor microenvironment.	No human data and brief period of intervention.	Promising anticancer potential via metabolic and immune modulation.	[89]
Experimental in vitro	HepG2 and Huh-7.	HuaChanSu (0.5, 1, 2, 4, 8, and 16 µg/mL).	Suppresses G6PD enzyme activity via downregulation of PLK1; restrains NADPH production.	Inhibits cell proliferation and viability.	No human trials.	Potential as an adjuvant therapy for liver cancer.	[90]
Experimental in vitro and in vivo	HepG2 and SK-HEP-1. H22 hepatoma-bearing mouse model.	HuaChanSu (0.5, 1, 2, 4, and 8 µg/mL in vitro; 4 and 8 mg/kg in vivo).	Inhibition of glucose uptake and glycolysis via suppression of Hexokinase-2; induces apoptosis and cell cycle arrest.	Inhibits cell proliferation and tumor growth.	No clinical translation yet.	Potential for targeting tumor metabolism and growth.	[91]
Experimental in vitro and in vivo	SMMC7721. H22 hepatoma-bearing mice.	Cinobufacini (1.85, 5.56, 16.67, 25, and 50 mg/mL in vitro; 10.28 mL/kg in vivo); Bufothionine (343.35 μg/kg in vivo; 3, 10, 30, 100, and 300 μM in vitro).	Inhibits JAK2/STAT3 signaling; induces autophagy and apoptosis.	Relieved tumor symptoms in vivo and inhibits cell proliferation.	No clinical translation yet.	Potential for targeting JAK2/STAT3 in liver cancer.	[92]
Experimental in vitro	HepG2.	Cinobufacini (0.005, 0.01, 0.05, 0.1, and 0.5 mg/mL).	Inhibits EMT through c-Met/ERK signaling pathway; downregulates N-cadherin and vimentin; regulation of MMP.	Reduces cell proliferation, migration, and invasion.	In vitro only.	Potential EMT-targeting strategy in HCC, especially against metastasis.	[93]
Experimental in vitro and in vivo	BEL7402. HepG2 and H22-tumor bearing mice.	Bufadienolides (components of Cinobufacini) (IC_50_ = 0.28 ± 0.05 μg/mL in vitro; 0.5, 0.75, 1.5, 2, and 3 mg/kg in vivo).	The mechanisms underlying the antitumor effects of bufadienolides have not been reported.	Inhibits tumor growth.	No mechanism-specific assays.	Facilitate further research in improving the therapeutic effects of cinobufacini injection.	[94]
Experimental in vitro	HepG2 and HLE.	Cinobufacini (1, 0.1, 0.01, and 0.001 mg/mL).	Activates Fas and mitochondrial apoptosis pathways.	Inhibits cell growth; cinobufacini + doxorubicin induces apoptosis more significantly compared with each substance alone.	Only in vitro.	Combining cinobufacini with chemotherapeutic drug could improve outcomes and quality of life in HCC patients.	[95]
Experimental in vitro	HepG2.	Cinobufacini (IC_50_ concentrations were 0.86 ± 0.03, 0.08 ± 0.01, and 0.04 ± 0.06 mg/L following treatment for 24, 48, and 72 h).	Suppresses mRNA and protein expression levels of topoisomerase I and II.	Reduces cell proliferation; induces apoptosis and cell cycle arrest.	Only in vitro with one cell line tested.	Promising candidate for inclusion in existing chemotherapy protocols.	[96]
Experimental in vitro	HepG2.	Cinobufacini (0.01, 0.05, and 0.1 mg/mL).	Morphological changes (disorganization of actin filaments; significant shrinkage and deep pores in the cell membrane, with larger particles and a rougher cell surface) detected via atomic force microscopy.	Inhibits cell viability and proliferation; induces cell cycle arrest and cytoskeletal destruction.	Only in vitro with one cell line tested.	Alternative method to detect early cytotoxic responses and better understand biophysical functions of HCC cells induced by cinobufacini.	[97]
Experimental in vitro	HepG2 and BEL7402.	Cinobufacini (IC_50_ of HepG2 cells at the times of 24, 48, and 72 h were 0.20, 0.08, and 0.03 mg/mL, while those of BEL7402 cells were 0.15, 0.06, and 0.02 mg/mL, respectively).	Mitochondrial-mediated apoptosis pathway (increase in the Bax/Bcl-2 ratio; release of cytochrome c; activation of both caspase-9 and -3, and cleavage of PARP).	Induces apoptosis; inhibits cell proliferation.	In vitro only.	It may boost the efficacy of current therapeutic strategies against HCC.	[98]

**Abbreviations:** 5-FU—5-Fluorouracil; Akt—Protein kinase B; AMPK—AMP-activated protein kinase; Bax—Bcl-2-associated X protein; Bcl-2—B-cell lymphoma 2; c-Met— Tyrosine-protein kinase Met; DNA—Deoxyribonucleic acid; EMT—Epithelial–mesenchymal transition; ERK—Extracellular signal-regulated kinase; FASN—Fatty acid synthase; G6PD—Glucose-6-phosphate dehydrogenase; HCC—Hepatocellular carcinoma; JAK2—Janus kinase 2; MEK—Mitogen-activated protein kinase kinase; MMP—Matrix metalloproteinase; mRNA—Messenger ribonucleic acid; NADPH—Nicotinamide adenine dinucleotide phosphate; PARP—Poly (ADP-ribose) polymerase; PI3K—Phosphoinositide 3-kinase; PLK1—Polo-like kinase 1; SREBP1—Sterol regulatory element-binding protein 1; STAT3—Signal transducer and activator of transcription 3; TYMS—Thymidylate synthase.

**Table 3:** Effects of cinobufagin-containing standardized drug cinobufacini/HuaChanSu in the intervention of liver cancer in human subjects: outcomes, limitations, and implications across different studies.

Population	Study Design	Intervention	Comparison	Outcomes	Limitations	Reference
Eleven patients with advanced HCC.	Pilot study, phase I trial design.	Intravenous cyclic HuaChanSu for 14 days, followed by 7 days off (1 cycle). Patients were divided into groups with doses of 10, 20, 40, 60, or 90 mL/m^2^.	There is no placebo or control group.	One patient with HCC had 20% disease regression after 11 months using 10 mL/m^2^ of HuaChanSu and patients with stable disease had quality of life improvement.	The lack of a placebo or controlled comparison between groups limits the generalizability of the findings.	[99]
364 patients aged between 18 and 75 years who underwent radical hepatectomy and had HCC diagnosed by histopathological examination.	Multicenter, randomized, open-label controlled trial.	Cinobufacini injection 50 mL per day for 10 days every 3 months for 12 months, associated with Jiedu granule 4.5 g twice a day for 6 months (THM formula).	Transarterial chemoembolization.	THM was associated with decreased risk of HCC recurrence after resection, with comparable adverse effects.	The study analyzed the THM formula as a whole entity, without deconstructing it into individual active ingredients.	[100]

**Abbreviations:** HCC—hepatocellular carcinoma; THM—Traditional herbal medicine.

## Data Availability

No new data were created or analyzed in this study.
